# Moderate pyridoxal phosphate deficiency enhances neuronal excitability and promotes calcium dysregulation

**DOI:** 10.3389/fnins.2025.1621349

**Published:** 2025-06-23

**Authors:** Valerie Girgis, Ashlyn Tharington, Jadyn Trivett, Sylvia Fitting, Rick B. Meeker, Clio Rubinos

**Affiliations:** ^1^Department of Psychology and Neuroscience, University of North Carolina at Chapel Hill, Chapel Hill, NC, United States; ^2^Department of Neurology, University of North Carolina School of Medicine Chapel Hill, Chapel Hill, NC, United States

**Keywords:** pyridoxine, pyridoxine deficiency, seizures, neuronal excitability and spiking, calcium dysregulation

## Abstract

**Objective:**

Pyridoxal 5′-phosphate (PLP), the active form of pyridoxine (vitamin B6), is essential for converting glutamate into the inhibitory neurotransmitter gamma-aminobutyric acid (GABA). Severe consequences of PLP deficiency due to genetic defects are well known, but the impact of modest pyridoxine deficits remains unclear. Low pyridoxine levels have been associated with poor neurological outcomes in disease and injury, but it is uncertain whether this reflects a causal relationship or is a secondary consequence of inflammatory conditions. This study aimed to investigate the functional impact of moderate reductions in PLP activity on neuronal function.

**Methods:**

Primary mouse neuronal cultures were treated with amino-D-proline (ADP) to induce a moderate reduction in PLP activity to simulate a low pyridoxine environment. ADP concentrations were titrated to produce a small decrease in GABA expression without loss of GABAergic cells or innervation. Calcium signaling was assessed in live cells, and electrophysiological recordings were performed using multielectrode arrays to evaluate neural activity under baseline and mild pathological conditions (reduced magnesium, inflammatory environment).

**Results:**

ADP-treated neurons exhibited increased calcium signaling frequency and intensity, consistent with a hyperactive phenotype. Under mild pathological conditions, calcium signaling and accumulation were further amplified. Electrophysiological recordings revealed increased neural activity, characterized by a higher frequency of short bursts of synchronous activity and random spikes. Chronic ADP treatment led to compensatory changes in neural activity, suggesting potential differences between acute and chronic pyridoxine deficiency.

**Conclusion:**

This study demonstrates that even a modest reduction in PLP activity induces transient neuronal hyperexcitability, which may enhance the pathological effects of disease, injury, and inflammation. These findings highlight the importance of pyridoxine in maintaining neural stability and suggest that low pyridoxine levels could contribute to neurological dysfunction. Further investigation is warranted to fully understand the clinical implications of mild pyridoxine deficiency.

## Introduction

Pyridoxal 5′-phosphate (PLP), the active form of Vitamin B6 (pyridoxine), plays a role in numerous enzymatic reactions in the body including transamination of amino acids, decarboxylation reactions, and regulation of gene expression (Amadasi et al., 2007; Ueland et al., 2017). In the central nervous system (CNS), PLP is an essential cofactor for glutamate decarboxylase (GAD), which decarboxylates glutamate into the inhibitor transmitter gamma-aminobutyric acid (GABA). Severe loss of PLP activity (and GABA) due to a rare autosomal disorder causes pyridoxine-dependent epilepsy, characterized by intractable seizures and intellectual disability. Supplementation of vitamin B6 (pyridoxine) is the treatment of choice (Baumeister et al., 1994).

While pyridoxine-dependent epilepsy results from severe PLP depletion due to a genetic mutation that disrupts pyridoxine metabolism, the clinical impact of pyridoxine deficiency in the absence of a genetic mutation remains less clear. Decreased production of GABA in the nervous system due to a PLP deficiency could increase brain hyperexcitability (Guilarte, 1989; Jung et al., 2019; Rane et al., 2022) and affect disease or brain injury outcome. Retrospective studies of patients with status epilepticus (SE) found low pyridoxine levels in 80–94% of patients (Dave et al., 2015; Rubinos et al., 2022). A comparison of established status epilepticus (eSE, benzodiazepine resistant SE) patients, to intensive care unit (ICU) patients without SE (ICU-noSE), non-ICU hospitalized patients without SE (non-ICU), and outpatients with or without epilepsy showed that the eSE group had lower pyridoxine levels, measured by PLP in serum, suggesting a relationship between PLP and seizure severity (Rubinos et al., 2022). Notably, pyridoxine deficiency (<20 nmol/L) prevalence in non-eSE and non-ICU patients was 50–54%, supporting the possibility that low PLP could be a significant risk factor in a variety of settings where strong excitatory activity contributes to neural dysfunction and damage (Rane et al., 2022).

Because pyridoxine is widely available in animal and plant derived foods, deficiency considered to be clinically relevant is not common (Paul et al., 2013). However, low pyridoxine is common, particularly in patients with inflammatory conditions such as rheumatoid arthritis, type 1 diabetes, obesity and HIV infection (Chiang et al., 2003; Chiang et al., 2005; Rubinos et al., 2022).

In addition to the suppression of GABA synthesis, pyridoxine deficiency can also influence neural activity through the kynurenine pathway. Under inflammatory conditions, degradation of tryptophan through the PLP-dependent indoleamine 2,3-dioxygenase (IDO) pathway, can affect the conversion of kynurenine to kynurenic acid or anthranilic acid, as well as the conversion of 3-hydroxykynurenine to 3-hydroxyanthralinic acid or xanthurenic acid. Mobilization and high consumption of PLP by the immune system in response to inflammation (Ueland et al., 2017; Paul et al., 2013; Lotto et al., 2011) reduces availability and the loss of kynurenic acid may shift metabolism to the production of quinolinic acid, a known excitotoxin (Paul et al., 2013). Thus, patients with underlying inflammation may have a higher risk of neuron overexcitation, contributing to the development of acute symptomatic seizures and disease progression due to the depletion of PLP in the CNS. However, because neurological diseases are often accompanied by inflammation, it has been difficult to determine if low PLP is instrumental in facilitating disease processes or simply an unrelated correlate due to depletion by inflammatory activity.

To determine if there is a direct impact of PLP depletion on neural function in the absence of inflammation, we created a modest PLP deficiency in primary mouse neurons by treating with the PLP inhibitor, amino-D-proline (ADP). Under these conditions, a moderate hyperexcitable neuronal phenotype developed which greatly increased the vulnerability of neurons in excitatory and inflammatory environments.

## Materials and methods

### Primary neural cell cultures

All work with animals followed the guidelines from the National Research Council’s Guide for the Care and Use of Laboratory Animals and was approved by the UNC Institutional Animal Care and Use Committee. Pregnant female C57/bl6 mice (Charles River) were euthanized in isoflurane at gestational day E16, the uterus was removed, rinsed briefly in 70% ethanol and then placed in sterile, ice cold HEPES-buffered Hank’s balanced salt solution (HBSS). The brain was removed from each fetus, washed 4X, stripped of membranes, the cortex/hippocampus removed and minced. The pieces were transferred to a tube containing 20 U/mL papain and 5–10 μL (10–20 U) DNase (Worthington). The tissue was incubated at 37° C for 30 min and gently triturated to facilitate dissociation of the cells. The suspended cells were then transferred to a culture tube containing 2 mL/harvested brain of Neurobasal Plus medium, B27 Plus supplement, glutamine, 5% fetal bovine serum, and 20 μg/mL gentamicin (plating medium). The dissociated cells were counted and seeded at a density of 12,000 to 14,000 cells/cm^2^ on coverslips previously treated with 0.1 mg/mL poly-D-lysine. The neurons were fed with fresh medium (Neurobasal Plus medium, B27 Plus supplement, 2 mM glutamine, and 20 μg/mL gentamicin) the following day and then 3 times a week with a 50% medium exchange. Neurons were used between 18 and 32 days in culture unless otherwise indicated.

### Treatment with amino-D-proline

**Figure 1:**
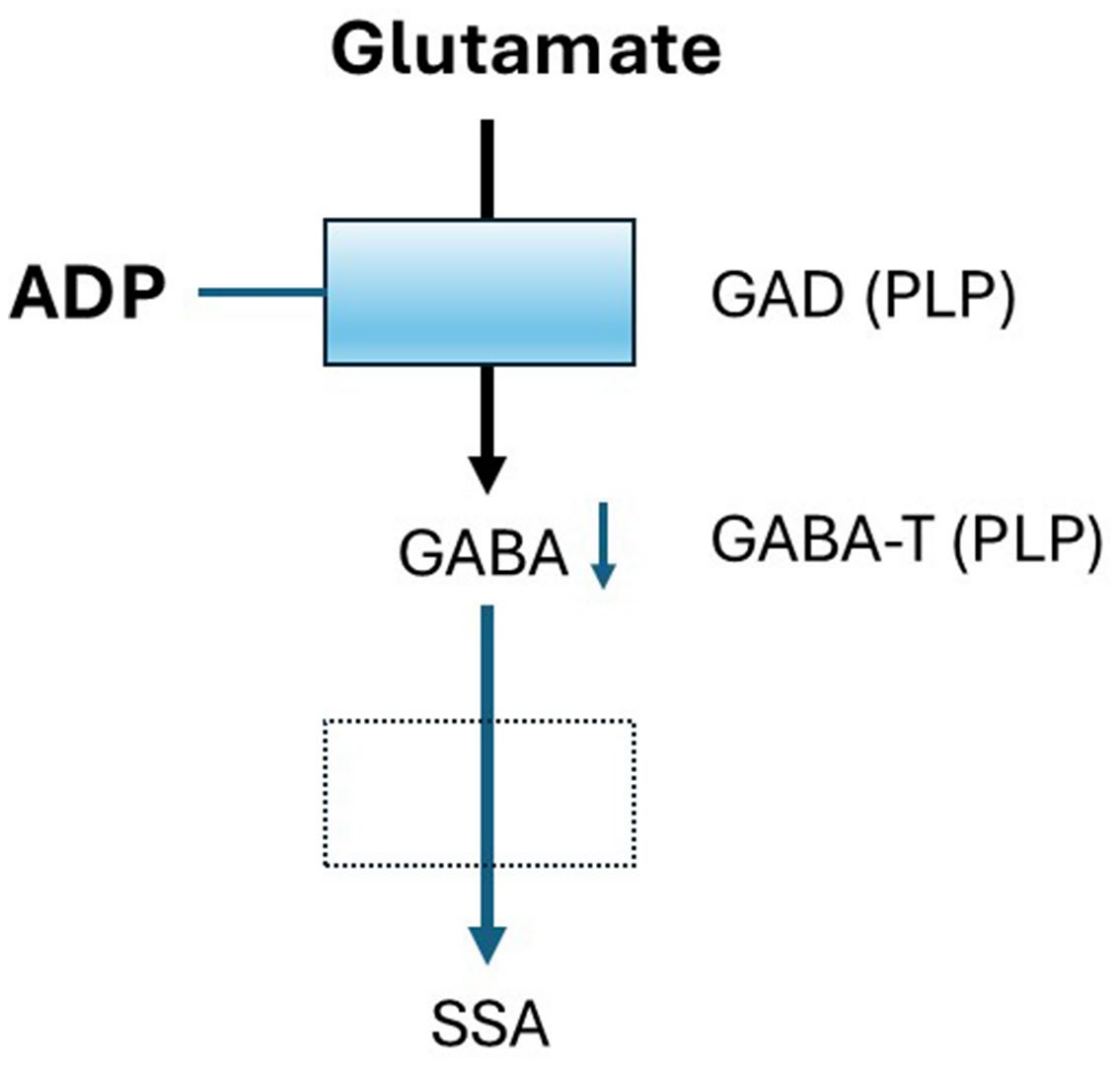
Mechanism of ADP Activity. Two enzymes in the Glutamate-GABA metabolic pathway are dependent on the cofactor pyridoxal phosphate (PLP), Glutamic acid decarboxylase (GAD) and GABA transaminase (GABA-T). ADP binds to PLP and inhibits it is activity in a concentration-dependent fashion resulting in decreased GABA synthesis. Using a low concentration of ADP results in a small loss of GABA that may mimic conditions in individuals with low pyridoxine. Effects on GABA-T are thought to be small relative to GAD.

ADP was obtained from Toronto Research Chemicals and prepared as a 10 mM stock solution in artificial cerebrospinal fluid (aCSF). ADP does not initiate an inflammatory cascade, instead, it binds to PLP, forming an inactive complex that inhibits PLP-dependent enzymes and depletes their related products, including those involved in GABA synthesis (Mayengbam et al., 2016; Figure 1).

To mimic moderate pyridoxine deficiency, defined as the level of inhibition that produces minimal reduction in GABA, ADP was titrated to produce a very small decrease in GABA expression with no observable toxicity. Toxicity was assessed on measurement of intracellular calcium and structural analysis of MAP-2 staining for neuronal structure. Concentrations ranging from 0 (vehicle, aCSF), 1, 3, 10, 30, and 100 μM were tested and 3.2 μM was identified as the optimal dose that produce a very small reduction of neuronal GABA with no measurable toxicity. This strategy was designed to model the subtle, subclinical effects of moderate pyridoxine deficiency that may exist in patients. While the exact threshold at which low pyridoxine impairs GABA synthesis in humans is unknown, this approach enables investigation into how early, modest reductions in GABA may increase neuronal susceptiblility to hyperexcitability.

### Multielectrode arrays

Axion 64 multielectrode arrays (MEAs) were used in a 6-well format. Electrodes were coated with a 20 μL bead of 0.05% of polyethylenimine (PEI) or 0.1 mg/mL poly-D-lysine and neurons were seeded from a suspension of 1.5 × 10^6^ cells/mL at an average density of ~150,000 cells/cm^2^. After the neurons attached, the MEAs were fed with 200–300 μL of plating medium and incubated overnight at 37° C with 5% CO_2_. Cultures were fed three times per week with Neurobasal Plus as indicated above. ADP was added at 20 days in culture after confirmation of mature network activity and neurons were recorded over 4 weeks with continuous exposure to ADP.

#### Analysis of multielectrode array (MEA) data

MEA data was collected at 12.5 kHz with a low pass filter of 3 kHz (Kaiser Window), a high pass filter of 200 Hz (IIR) and coincident event removal. Spikes were identified using an adaptive threshold and a cutoff of 6 standard deviations above baseline. A 10 min recording was collected after equilibration of temperature and CO_2_ and a minimum spike rate of 5/min was used to identify active electrodes. Burst detection required a minimum of 35% participating electrodes. A stringent maximum interspike interval (ISI) was set at 24 ms to capture sequential bursts that occasionally appeared in rapid succession. Axion Axis Navigator and Neural Metrics software were used for analysis of data from 3 to 6 MEAs representing up to 384 electrodes.

### Intracellular calcium imaging

Live cell imaging of neuronal intracellular calcium was measured in aCSF (aCSF: 127 mM NaCl, 5.0 mM KCl, 2.3 mM CaCl2, 1.3 mM MgCl2, 20 mM glucose) after loading the neurons for 30 min in aCSF containing the fluorescent calcium indicator Fluo-4 AM (2 μM, Molecular Probes Inc., Eugene, OR). Fluorescence changes were measured on an Olympus IMT-2 inverted microscope at a final magnification of 674x. To establish baseline calcium levels, cells were imaged every 6 s for 6 min with a 40 ms exposure and a 0.5 neutral density filter using Metamorph software (Molecular Devices, Downington, PA). Neurons exposed to the inflammatory environment were imaged every 6 s for 6 min (acute phase) followed by every minute for 40 min (sustained calcium dysregulation). Fluorescence brightness was quantified in individual neurons using Metamorph image analysis software. Average calcium levels as well as individual neuronal calcium spikes were quantified to indicate calcium homeostasis/dysregulation (intracellular calcium accumulation) and intercellular communication (calcium spikes).

To identify individual calcium signaling events (spikes), a threshold was set at 3.2 standard deviations above basal calcium levels (*p* = 0.001) for each run.

Chronic intracellular calcium accumulation was measured at each time point as the average calcium level for all neurons in a given field. To determine changes in calcium homeostasis, the ADP acute calcium peak and the delayed calcium maximum accumulation were averaged across neurons and compared to the vehicle.

### PLP inhibition under conditions of enhanced neural activity

#### Low magnesium

While low pyridoxine alone may not have clinically significant effects, it may synergize with other conditions such as those that enhance glutamatergic signaling. To test whether PLP inhibition with chronic ADP treatment would synergize with increased N-methyl-D-aspartate (NMDA) activity, we decreased the magnesium concentration of the aCSF to 0.1 mM to reduce the magnesium block of the NMDA receptor. Calcium changes were quantified as indicated above.

#### Inflammatory challenge

To determine if inhibition of PLP might synergize with conditions induced by an inflammatory stimulus, we exposed ADP treated neurons to a very mild inflammatory environment. Medium produced from human macrophages that were treated with 1 μM Aβ oligomers (macrophage conditioned medium, MCM) was used to mimic inflammatory conditions present in Alzheimer’s disease. At a dilution of 1:5 the MCM produces a strong dysregulation of intracellular calcium, chronic elevation of intracellular calcium and cytoskeletal damage (Tseng et al., 2017; Meeker et al., 2016). To minimize the inflammatory challenge and optimize detection of a synergistic response, we identified a very mild challenge by performing a dilution series of the MCM at 1:10, 1:20, and 1:25. A 1:25 dilution provided a minimally detectable effect on calcium dysregulation (4.0% of acute and 12.6% of delayed calcium at 1:10) and was used in the assays to determine synergism with ADP treatment (Supplementary Figure 1).

This experimental design using MCM was intentional to ensure that any observed increase in neuronal hyperexcitability could be attributed to the synergistic interaction between ADP-induced PLP inhibition and a subtle inflammatory stimulus, rather than to inflammation alone. While TNF *α* and IL-1β are important markers of inflammation activity, alone do not reproduce the full effects of inflammation when applied directly to neurons. Given this complexity, we used what we believe to be the most representative surrogate of the true inflammatory environment.

### Immunostaining

Immunostaining with microtubule-associated protein 2 (MAP-2) was used to provide a detailed picture of neuronal morphology and early signs of cytoskeletal pathology. Rabbit or mouse anti-MAP-2 (Millipore) was used at a dilution of 1:500 followed by an Alexa Fluor 488 conjugated goat anti-rabbit/mouse (1:500). To visualize GABAergic neurons, mouse anti-GAD67 (Millipore MAB 5406, 1:500) or rabbit anti-GABA (Sigma, A2052, 1:500) was coupled to secondary antibodies conjugated with Alexa Fluor 594 (1:500). Nuclei were stained with 0.5 μg/mL bisbenzimide.

Cultured neurons were fixed in a solution of 2% paraformaldehyde for the GABA/MAP-2 stain and 97% methanol/3% acetic acid for the GAD67/MAP-2 stain. The cells were washed in phosphate buffered saline (PBS) 3 × 5 min, blocked with 3% normal goat serum blocking (1 h) and then incubated 1–2 days in primary antibodies. The cells were washed 3X and incubated in Alexa594 secondary antibody for 1 h. The cells were washed 3X, counterstained with bisbenzimide, washed 3X and mounted onto slides with Fluoromount.

#### Identification of GABAergic cells and GABA expression

GABAergic cells were counted manually and verified by automated analysis of cells thresholded for intensity using Metamorph software. Stained objects were sorted by size and the number of large objects (neurons) was counted as well as neuronal processes and small synapse-like varicosities. Five images were collected and analyzed for each culture.

## Results

**Figure 2:**
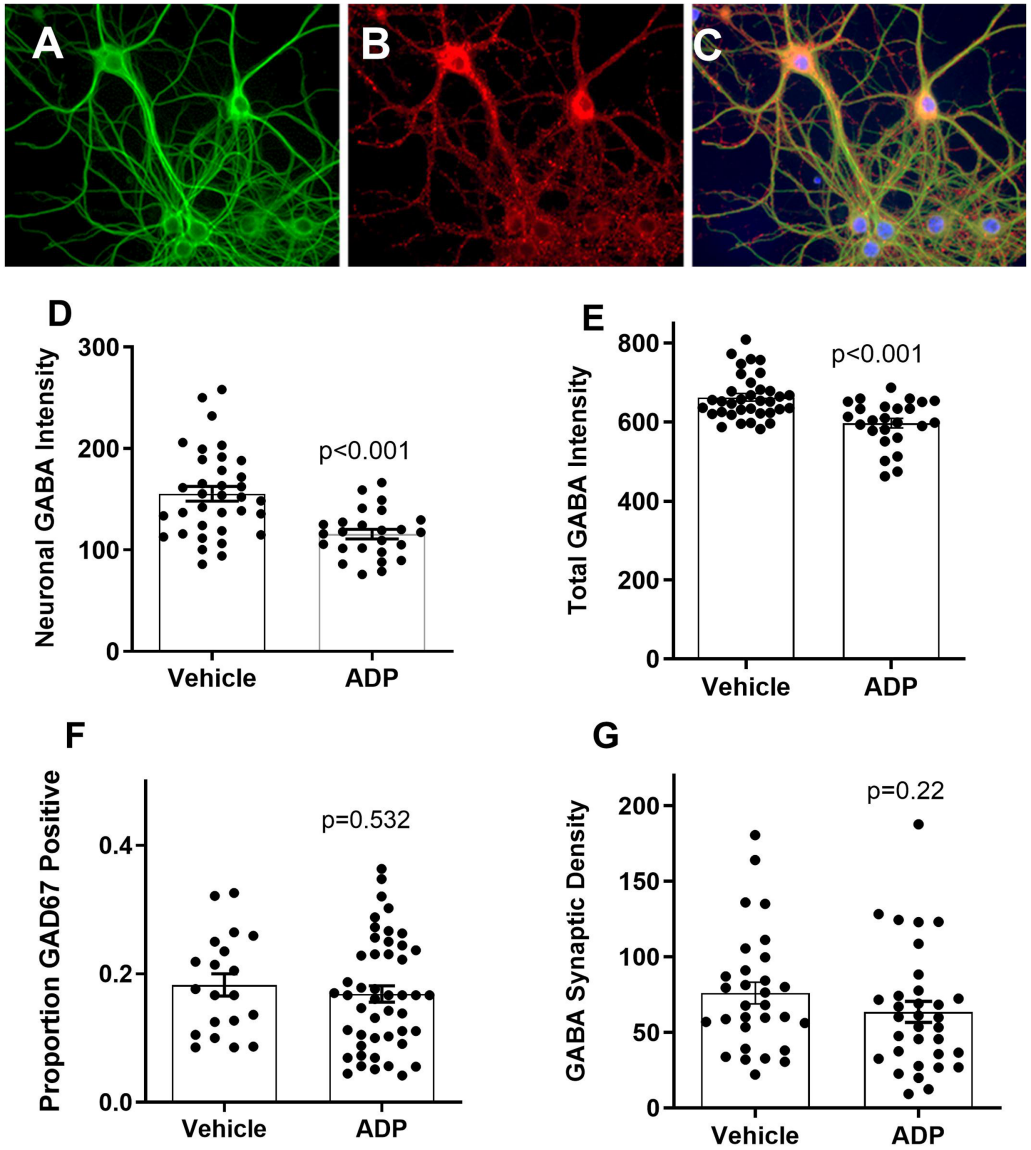
Example of neurons at 24 days in culture stained for MAP-2 (green), GAD67 (red), and nuclei (bisbenzimide, blue). MAP-2 staining **(A)** showed normal neuronal morphology under all conditions. Two GAD67 positive cell bodies **(B)** are bright red relative to five negative neurons at the bottom. Small punctate red varicosities, reflecting presynaptic boutons, were readily visible on all cells indicating extensive GABAergic innervation **(C)**. Cultures treated with ADP for 7 days have decreased expression of neuronal cell body **(D)** and total GABA **(E)** (t-tests, *p* < 0.001). There was no significant difference in the relative number of GABAergic cells **(F)** based on the GAD67 stain or the density of synaptic-like varicosities **(G)**.

Treatment with 3.2 μM ADP caused no overt differences in neuronal morphology (Figure 2A) or GABA expression (Figures 2B,C). Quantification of neuronal GABA expression revealed a modest 25.5% reduction (Figure 2D) and a smaller 9.8% reduction in total GABA expression (perikarya, processes and terminals; Figure 2E). No significant change in the proportion of GAD67-positive neurons (17–18%) (Figure 2F) and synaptic density were seen (Figure 2G), indicating that the decrease in GABA was not due to a loss of GABAergic cells or terminals. Thus, under these conditions, ADP treatment had a very modest effect on GABA reflecting what might be expected under conditions of pyridoxine deficiency.

### ADP increased the frequency and intensity of basal calcium signaling

**Figure 3:**
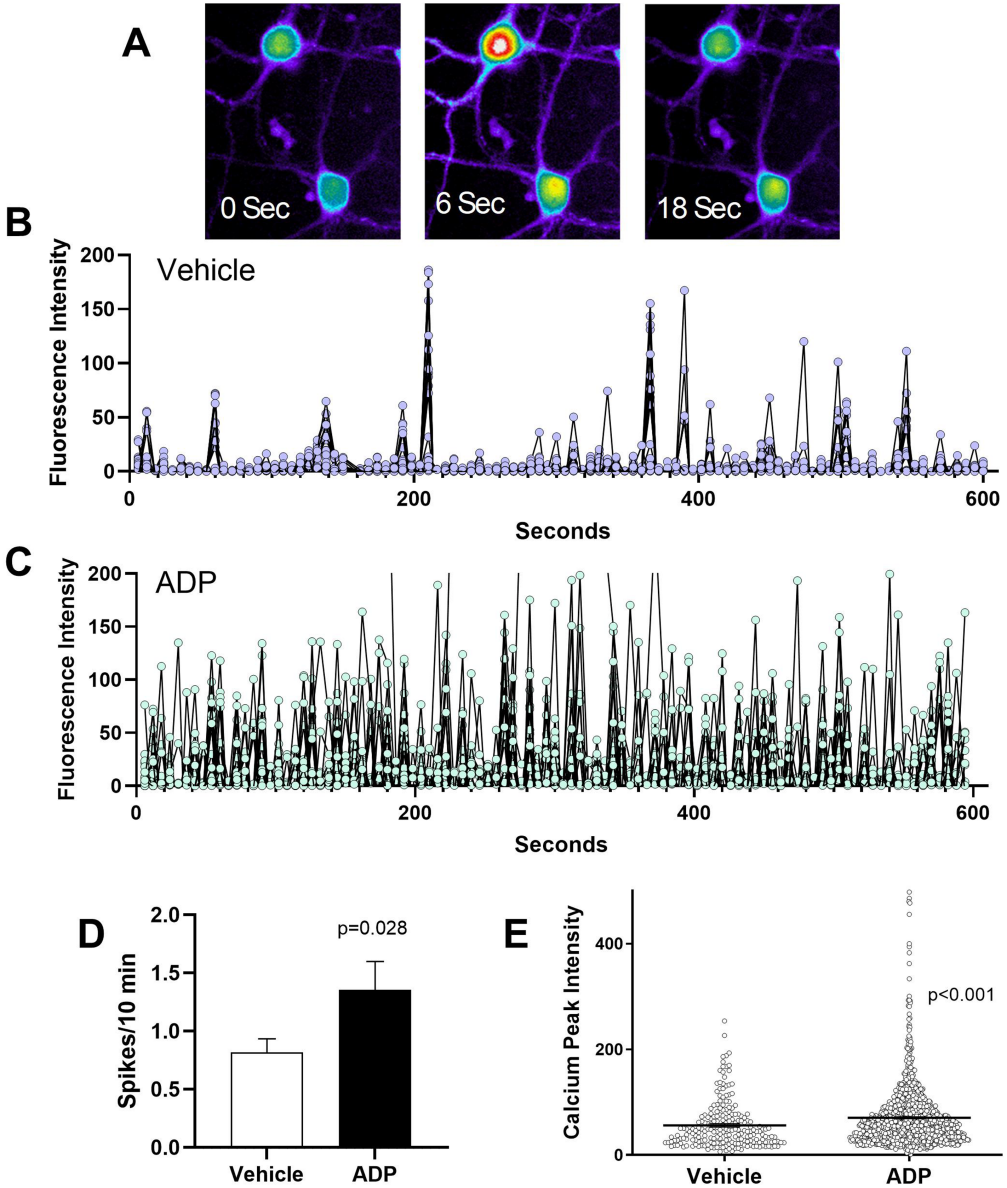
ADP intensifies basal calcium signaling. **(A)**. Sequential images of two Fluo4 loaded neurons showing calcium levels at 0 s, before a neuronal calcium spike, during the neuronal calcium spike (6 s), and 12 s after the calcium peak. Calcium levels are pseudocolored to show the intensity of the signal (highest to lowest: white > red > yellow > green > blue). **(B,C)** Illustration of basal calcium signaling over a 10 min period for a composite of 30 representative vehicle control neurons **(B)** and 30 representative ADP-treated neurons **(C)** sampled from 3 typical runs each. Neurons treated with ADP exhibited an increased frequency and intensity of calcium signaling. **(D)** Average calcium spikes/10 min across 9–12 experiments showed consistently higher spike frequency for ADP treated neurons (*t*-test, *p* = 0.028; 110 neurons) relative to vehicle controls (104 neurons). **(E)** Calcium signaling (peak calcium intensity) was significantly stronger in ADP treated neurons relative to vehicle controls (*t*-test, *p* < 0.001).

Figure 3A illustrates a typical calcium spike. The frequency and intensity of intrinsic calcium spikes was measured in resting neurons. Typical spiking patterns over a period of 10 min in 30 neurons treated for 7 days with ADP or vehicle are illustrated in Figures 3B,C. Relative to controls (Figure 3B), neurons treated with ADP (Figure 3C) have an increased number and intensity of calcium spikes. In the vehicle control, the average calcium spike rate (Figure 3D) was 0.82 spikes/10 min with an average peak intensity of 56.1 fluorescence brightness units (Figure 3E), while ADP-treated neurons have 65% greater spike frequency (1.35 spikes/10 min, Figure 3D) and averaged 70.6 fluorescence brightness units (Figure 3E).

### ADP sensitizes NMDA-dependent calcium signaling

**Figure 4:**
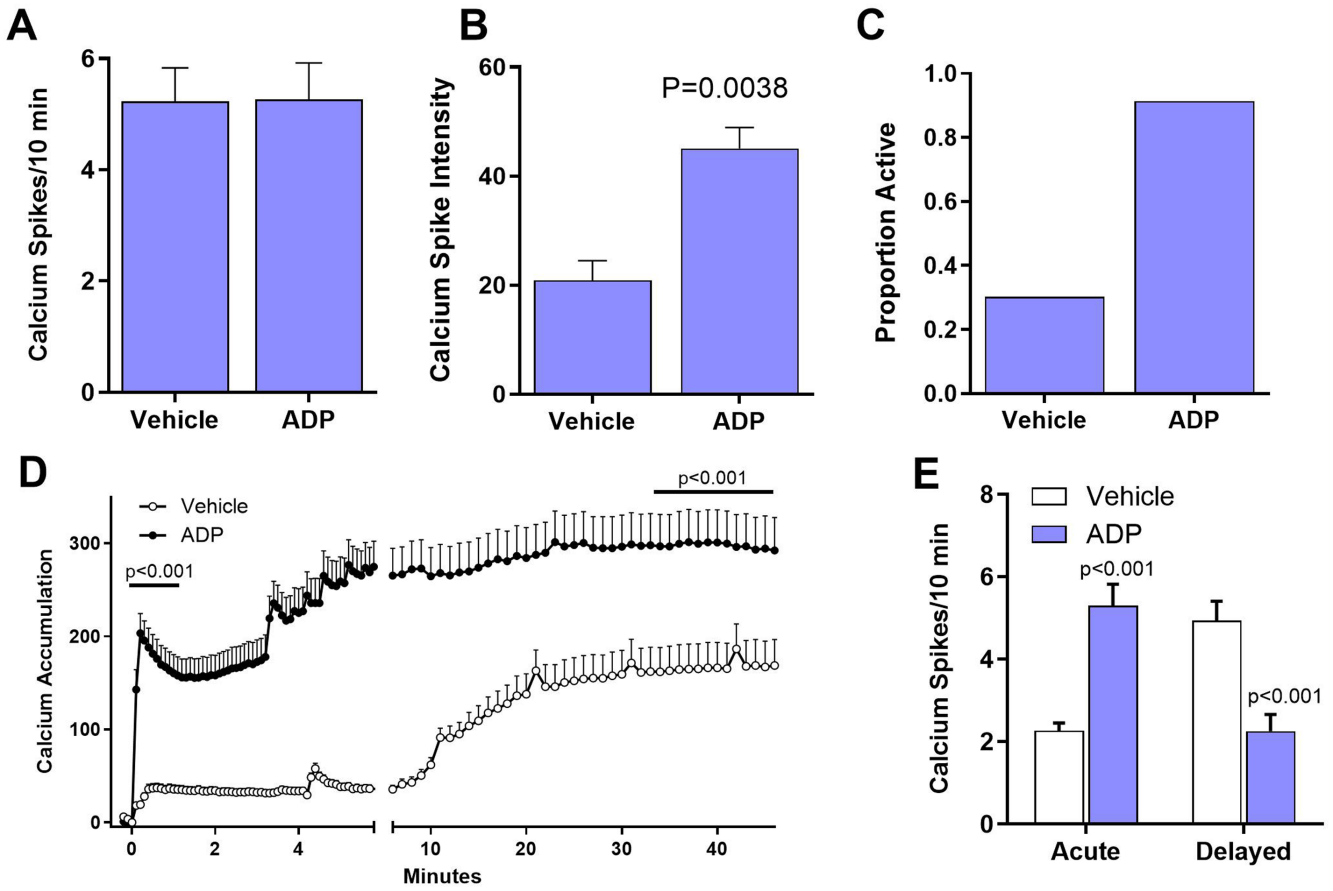
ADP enhances NMDA and inflammatory pathology. **(A)**. The average number of calcium spikes was greatly enhanced in 0.1 mM magnesium but was the same for ADP and vehicle controls. In each case, inactive cells (0 spikes) were not included in the averages. **(B)** Calcium spike intensity was significantly higher for ADP treated cells (*t*-test, *p* = 0.0038). **(C)** The proportion of active cells was higher in the presence of ADP. **(D)** After treatment with MCM (1:25) to simulate a mild inflammatory environment, ADP treatment resulted in a 4.7-fold larger acute dysregulation of calcium relative to matched vehicle neurons (average of first minute post-stimulation, *p* < 0.001). Delayed dysregulation of calcium was also significantly higher in ADP (average of last 10 min, *p* < 0.001). **(E)** Neurons treated with ADP (*n* = 62 neurons, 4 experiments) showed an increased average number of calcium spikes (*t*-test, *p* < 0.001) in the acute phase relative to vehicle (*n* = 104 neurons, 4 experiments), but a decreased average number of spikes in the delayed phase (*t*-test, *p* < 0.001).

We hypothesized that partial relief of the magnesium block of the NMDA receptor by reducing magnesium in the aCSF to 0.1 mM would lead to exaggerated neuronal excitability in the ADP treated neurons. The average number of spikes per neuron (5.2 spikes/10 min) was increased relative to basal activity (0.8 spikes/10 min) for both vehicle and ADP (Figure 4A). Thus, although the reduced magnesium had the expected effect on spike frequency, there was no synergistic effect of ADP. However, the intensity of spikes was 2.2-fold greater in the ADP-treated cells (Figure 4B) relative to vehicle controls. In addition, the proportion of active cells was 3-fold greater in the presence of ADP (Figure 4C) suggesting that “inactive” neurons become active under conditions of reduced GABAergic inhibition. The enhanced calcium signaling and increased probability of neuronal signaling are consistent with the hypothesis that low PLP activity enhances excitability without affecting average spike frequency.

### ADP enhances calcium accumulation under inflammatory conditions

The average time course of calcium accumulation under mild inflammatory conditions (1:25 MCM) is illustrated in Figure 4D. For vehicle control neurons, there was a very small acute increase in intracellular calcium (Figure 4D), followed by a small, gradual delayed rise. Relative to vehicle, the ADP-treated neurons, showed a 5-fold greater acute response and an 83% increase in delayed accumulation. The ADP treated neurons also had significantly greater calcium spikes/10 min in the acute phase but a decreased number of spikes in the delayed phase (Figure 4E). Since the enhanced calcium accumulation in ADP could be due, in part, to inefficient recovery (Meeker et al., 2005), we examined calcium recovery kinetics. Recovery was only slightly increased in ADP (Supplementary Figure 2) indicating that the increases in intracellular calcium were largely due to hyperactivity.

### ADP effects on neural activity

To determine how the cellular hyperexcitable phenotype translated to network activity, neurons cultured on multielectrode arrays were treated with 3.2 μM ADP at 20 days in culture (DIC). Activity was monitored over a period of 37 days with continuous exposure to ADP (57 DIC) followed by several weeks of monitoring after washout.

#### ADP caused transient increases in neural activity

**Figure 5:**
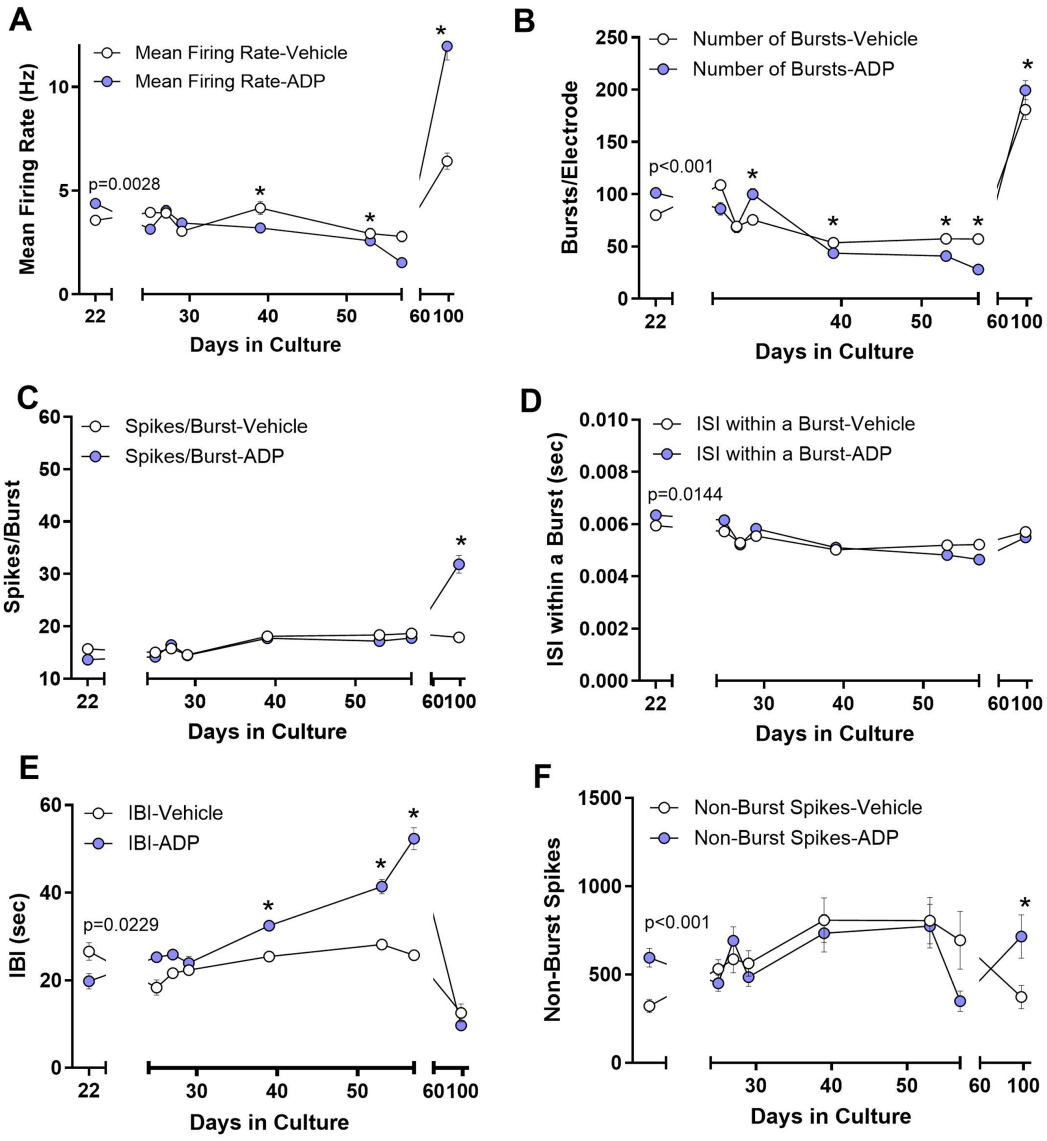
Acute (Day 22) and long-term (Days 24–56) changes in neural activity during treatment with 3.2 μM ADP. **(A)** Mean firing rate increased during initial exposure to ADP (2 days, t-test, *p* = 0.0028) and then showed a gradual adaptive decrease after about 19 days in ADP (39 DIC). These effects reversed after ADP washout. *p < 0.05 for time points during long term exposure. **(B)** The number of bursts/electrode increased in ADP (*t*-test, *p* < 0.001) followed by an adaptive decrease after about 19 days. A small reversal was seen for ADP versus vehicle after washout. **(C)** The average number of spikes/burst did not change with acute or chronic ADP but showed an increase after ADP washout. **(D)** The average ISI within a burst was very slightly increased acutely (*t*-test, *p* = 0.0144) followed by a return to control levels. No differences were seen for ADP versus vehicle after washout. **(E)** The interburst interval (IBI) initially decreased in ADP (*t*-test, *p* = 0.0229), consistent with the increased number of bursts, and then increased progressively over time after about 19 days in ADP. No differences were seen for ADP versus vehicle after washout. **(F)** Isolated spikes occurring outside of the bursts increased acutely (*t*-test, *p* < 0.001) and then returned to control values after 5 days in ADP. A small increase was seen for ADP versus vehicle after washout. Data are the mean ± SEM summarizing results from 3 to 5 MEAs per condition. Washout summary data represents the average of four consistent MEA runs from 88 to 106 DIC.

At 22 DIC, (2 days in ADP), there was a 27.0% increase in the mean firing rate (Figure 5A), a 26.4% increase in the average number of bursts/electrode (Figure 5B), and a small increase in intraburst ISI (Figure 5D) with no change in average spikes/burst (Figure 5C) or burst duration (not shown). A decrease in the interburst interval (IBI, Figure 5E) was consistent with the increase in bursts/electrode. An 85% increase in random non-burst spikes was also seen (Figure 5F). While these early effects were consistent with results from pharmacological inhibition of GABA_A_ receptors (Eisenman et al., 2015; Lonardoni et al., 2017; Jimbo et al., 2000; Darbon et al., 2002), subsequent recordings after 5 days in ADP began to show a rebound, similar to observations in previous studies of homeostatic plasticity (Turrigiano and Nelson, 2004; Wenner, 2011).

#### Chronic exposure to ADP suppressed neural activity

By 39 DIC (19 days in ADP) neurons showed consistently lower mean firing rates (Figure 5A), decreased bursts/electrode (Figure 5B), a small increase in intraburst ISIs (Figure 5D) and an increase in the IBI (Figure 5E), with no significant difference in active electrodes (83.8 vs. 86.4%) and number of spikes/burst (Figure 5C).

#### Elevated neural activity after ADP washout

Previous studies of neurons exposed to GABAergic blockade with 4-AP/bicuculline for 24 h show rebound effects (decreased activity) 4 h after removal of the drug (Vincent et al., 2013) indicating adaptation to the loss of GABAergic inhibition. When we removed ADP after 37 days of chronic exposure (59 DIC), a sustained increase was seen in the mean firing rate (Figure 5A), bursts/electrode (Figure 5B), spikes/burst (Figure 5C) and non-burst spikes (Figure 5F). These findings indicate an unexpected, increase in activity with long-term exposures.

## Discussion

### ADP treatment increased basal neural activity

While it is well established that a complete loss of PLP activity due to a genetic defect results in severe super-refractory seizures, it is unclear if a modest PLP deficiency under conditions of low pyridoxine might put neurons at risk for disease or damage associated hyperexcitability. Retrospective studies have clearly shown a correlation between low pyridoxine and neural damage but the high consumption of pyridoxine under conditions of inflammation make it difficult to determine if the low pyridoxine contributed to disease progression or was simply a secondary consequence of the disease or injury. A comparison of pyridoxine levels between patients with eSE and patients in the ICU without eSE revealed that eSE patients had significantly lower pyridoxine levels. This difference remained significant even after adjusting for critical illness severity, using Acute Physiology and Chronic Health Evaluation II (APACHE-II) score (Rubinos et al., 2022). The relationship between low pyridoxine and seizure activity could indicate that the low pyridoxine is a predisposing factor for seizure generation or the possibility that SE triggers time-dependent microglial activation that further enhances neuronal hyperexcitability and sustains seizure activity (Vezzani and Granata, 2005). In either case, low pyridoxine could contribute significantly to disease.

This conclusion was supported by the current study where a mild loss of PLP activity facilitated pathological neural activity under basal, excitatory and inflammatory conditions. Importantly, the effects of ADP were seen with no changes in the structure and health of the neurons and the proportion of GABAergic neurons and endings.

Although neurons treated with ADP showed only a slight reduction in total GABA (9%), there was an increase in the number and intensity of calcium spikes, indicative of a resting hyperexcitable phenotype. These findings support the hypothesis that a modest deficiency of PLP activity would pose a risk for toxic hyperexcitability due to a loss of inhibitory activity (Paul et al., 2013). Although the impact of pyridoxine deficiency on GABA production in humans is not known, these results illustrate that even a very small loss of GABA can alter the sensitivity of neurons.

The effect of ADP on glutamatergic neurons was not explored in this study. However, given the close interplay between glutamate and GABA, it is challenging to distinguish direct effects on glutamatergic neurons from those resulting from disinhibition in our model. While we proposed a simple hypothesis based on GABA loss, it is important to note that the effects of pyridoxine are inherently complex, as it influences multiple pathways – including the glutamate pathway.

### ADP enhanced NMDA receptor signaling

As discussed by Dave et al. (2015) and Rubinos et al. (2022), low levels of pyridoxine are common and correlate with hyperexcitability in individuals with neurodegenerative disease or acute brain injuries, such as a traumatic brain injury (TBI). To evaluate potential interactions of decreased PLP activity with conditions that promote excitability, we reduced the magnesium concentration from 1.3 mM to 0.1 mM to relieve the magnesium block in the NMDA receptor channel. As expected, neurons in low magnesium increased the rate of calcium signaling over 6-fold in both vehicle and ADP treated neurons. Although the signaling rate was the same in each condition, the average calcium spike intensity was doubled in the ADP treated cells and a greater proportion of neurons were active, consistent with a synergism between reduced GABAergic activity and enhanced NMDA receptor sensitivity. This synergism would be expected to increase the likelihood of calcium overload and make the brain more seizure-prone, particularly under conditions where low pyrixodine would be accompanied by increased glutamate release and/or reduced reuptake (Lewerenz and Maher, 2015).

### ADP treatment greatly increased calcium accumulation under inflammatory conditions

Almost all neurodegenerative conditions, including chronic diseases and acute injuries, are associated with inflammation (Heneka et al., 2015; Heppner et al., 2015; Perry et al., 2010; Zheng et al., 2022; Risbrough et al., 2022; Jacquens et al., 2022; Johnson et al., 2023). To determine how PLP deficient neurons might respond under conditions of inflammation, we modeled a weak inflammatory environment by adding dilute conditioned medium from Aβo challenged macrophages. While vehicle treated neurons showed minimal responses to the challenge, ADP treated neurons had a large acute increase in calcium followed by a delayed accumulation reaching levels that begin to produce cytoskeletal damage (Meeker et al., 2016). Thus, modest inhibition of PLP, increased the risk of neurons developing toxic levels of intracellular calcium.

While the reduction in calcium spikes in the delayed phase seems inconsistent with the large buildup of calcium, several studies have shown that as intracellular calcium levels rise above a certain pathological level (~250–300 fluorescence units in our experiments), opening of the NMDA and IP3 receptor channels is inhibited (Carter and Ogden, 1997; Bezprozvanny et al., 1991; Combettes et al., 1994; Finch et al., 1991; Iino, 1990; Kyrozis et al., 1996; Legendre et al., 1993; Rozov and Burnashev, 2016) resulting in less efficient signaling. Thus, by contributing to the calcium accumulation, low pyridoxine *in vivo* would be expected to enhance loss of function in degenerative diseases and in the chronic stages of brain injuries, potentially hindering neurorecovery following acute brain injuries.

### Neural network activity in ADP

The effects of loss of GABAergic inhibition on neural network activity has largely been studied following a total acute blockade of GABA_A_ receptors with bicuculline. Studies with MEAs have shown that GABA_A_ receptor blockade leads to calcium-dependent synchronous firing of hippocampal neurons (Penn et al., 2016), an increase in the mean firing rate and either a decrease (Lonardoni et al., 2017) or increase (Eisenman et al., 2015) in burst duration. In relatively young neural cultures (14 DIC), treatment with bicuculline/cyclothiazide induced an increase in the mean firing rate, due largely to an increase in the intraburst firing rate and number of bursts (Eisenman et al., 2015). Similarly, bicuculline/picrotoxin facilitated rhythmic electrophysiological and calcium activity in spinal cord neurons (Darbon et al., 2002). Notably, a decrease in excitability was seen at high intracellular calcium concentrations similar to the suppression of calcium spikes following calcium accumulation in our inflammatory model.

In our study, a small loss of GABA also resulted in an increase in the mean firing rate, number of bursts and enhanced calcium signaling indicating that even a small perturbation of GABA can significantly affect function. In contrast to the acute effects, chronic exposure to ADP, intended to mimic persistent, low pyridoxine, led to a sustained decrease in mean firing rate, bursts and spikes/burst. The burst duration and within burst ISIs were unaffected, indicating that the changes in network activity were not due to alterations in burst properties but rather a decrease in the ability to generate successive bursts. The findings suggest that individuals experiencing an acute decrease in PLP activity may be more prone to develop hyperexcitability than those with chronic deficiency where homeostatic plasticity may have down regulated excitatory activity.

Only a few studies have examined the long-term effects of GABAergic suppression. Pre-conditioning of neurons with 48 h exposure to bicuculline has subsequent protective effects due to a rebound suppression of excitotoxicity in some disease and injury models (Vincent et al., 2013; Tauskela et al., 2021). However, during washout after long-term exposure for weeks, we observed an increase in mean firing rates, longer burst durations, shorter IBIs and increased non-burst spikes. These changes persisted for weeks indicating that adaptive changes during long-term exposure to ADP were relatively stable. The physiological significance of these adaptions may indicate a neuronal network learning process that results in a persistent state of hyperexcitability, similar to the kindling effect. In this phenomenon, synaptic plasticity induced by repeated low-level electrical stimulation leads to a lasting increase in seizure susceptibility. Additional studies are needed to evaluate the effects of chronic pyridoxal phosphate reduction.

## Conclusion

Our data provide direct evidence that a small suppression of GABA due to PLP inhibition can facilitate disease processes. A hyperexcitable phenotype developed in neurons which synergized with NMDA receptor activity and development of calcium overload under inflammatory conditions. These effects highlight the potential vulnerability of neurons in individuals with concurrent pyridoxine (vitamin B6) deficiency. At the network level, neurons displayed a hyperactive phenotype characterized by an increase in mean firing rate, number of short bursts and non-burst spikes. During chronic inhibition, the neurons adapted to the ADP by gradually shifting to lower mean firing rates and fewer synchronous bursts with little effect on non-burst spikes. Evidence of sustained adaptation was seen after removal of the ADP where a relatively large increase in the mean firing rate, the burst duration and the ability to generate bursts was observed. Thus, low PLP activity can be an important factor influencing disease progression and further studies are needed to characterize the relationship between low vitamin B6/PLP and neuronal vulnerability.

## Data Availability

The original contributions presented in the study are included in the article/Supplementary material, further inquiries can be directed to the corresponding author.

## Supplementary material

Supplementary Figure 1 Mimicking mild inflammatory conditions with dilute macrophage conditioned medium (MCM). Human macrophages were activated with oligomeric Aβ (1 µM) and the medium was collected, centrifuged at 1000 x g and filtered at 300 kDa. The MCM, containing secreted factors that mimic the in vivo inflammatory environment, was then added to neuronal cultures at various concentrations and changes in intracellular calcium regulation were measured. At low dilutions (1:5, 1:10) the MCM induced a robust acute calcium response followed by a progressive delayed rise in calcium reflecting the inability of the cell to maintain calcium homeostasis. The large delayed rise in calcium is accompanied by cytoskeletal damage. At a dilution of 1:25, used in the current experiments, the acute and delayed calcium responses are just detectable, consistent with a very mild inflammatory environment.

Supplementary Figure 2 ADP-treated neurons show a small acceleration of calcium recovery. **(A)** The average calcium spike profile is similar for vehicle (*n* = 163 spikes) and ADP (*n* = 882 spikes) treated neurons. **(B)** Analysis of the recovery rate indicated an enhanced rate of recovery in neurons treated with ADP –0.0373 vs –0.0232 sec-1 (t test, *p* = 0.0016). Small calcium signaling events with less than three recovery data points were excluded from the analysis since accurate slope estimates could not be obtained and represented 23.8% and 22.4% of all spikes for vehicle and ADP, respectively.
