# Supplementary figures and images for: Moderate pyridoxal phosphate deficiency enhances neuronal excitability and promotes calcium dysregulation
Source: Front Neurosci. 2025 Jun 23;19:1621349. doi: 10.3389/fnins.2025.1621349 (PMC12229845; doi:10.3389/fnins.2025.1621349)

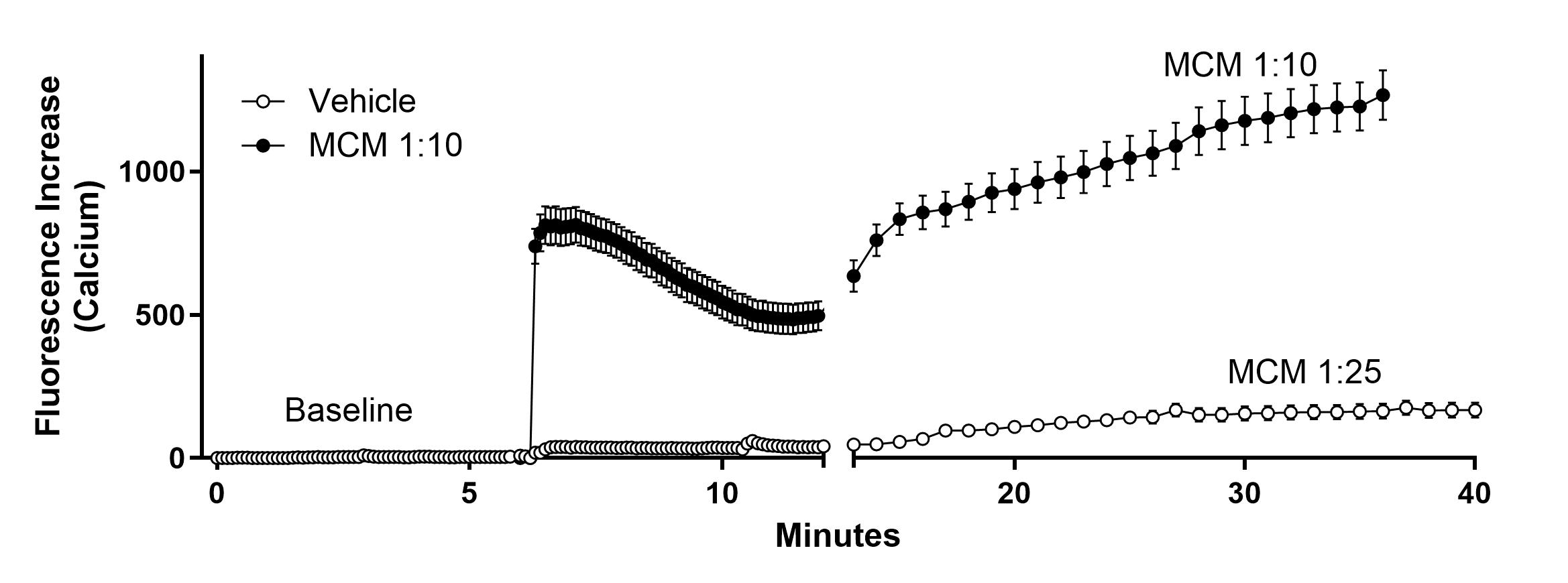

Supplement: Supplementary Figure 1 — Mimicking mild inflammatory conditions with dilute macrophage conditioned medium (MCM). Human macrophages were activated with oligomeric Aβ (1 µM) and the medium was collected, centrifuged at 1000 x g and filtered at 300 kDa. The MCM, containing secreted factors that mimic the in vivo inflammatory environment, was then added to neuronal cultures at various concentrations and changes in intracellular calcium regulation were measured. At low dilutions (1:5, 1:10) the MCM induced a robust acute calcium response followed by a progressive delayed rise in calcium reflecting the inability of the cell to maintain calcium homeostasis. The large delayed rise in calcium is accompanied by cytoskeletal damage. At a dilution of 1:25, used in the current experiments, the acute and delayed calcium responses are just detectable, consistent with a very mild inflammatory environment. [file Image_1.JPEG]

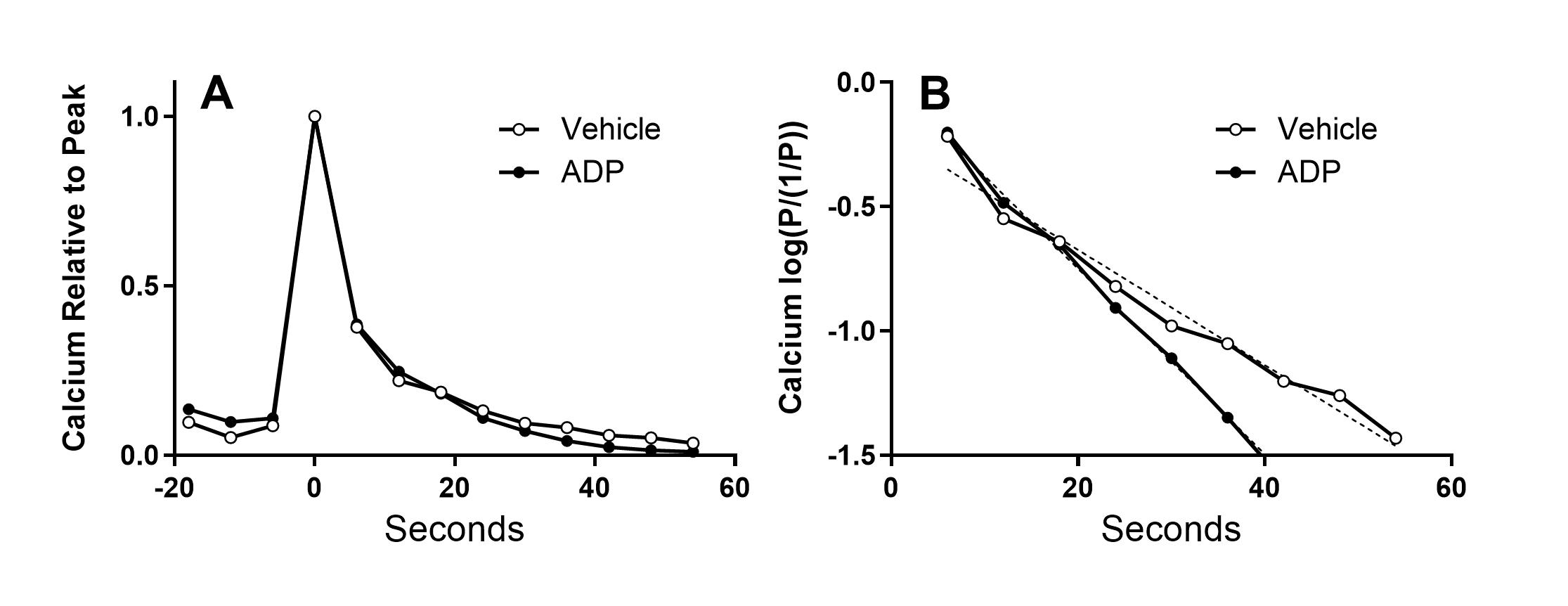

Supplement: Supplementary Figure 2 — ADP-treated neurons show a small acceleration of calcium recovery. (A) The average calcium spike profile is similar for vehicle (n = 163 spikes) and ADP (n = 882 spikes) treated neurons. (B) Analysis of the recovery rate indicated an enhanced rate of recovery in neurons treated with ADP –0.0373 vs –0.0232 sec-1 (t test, p = 0.0016). Small calcium signaling events with less than three recovery data points were excluded from the analysis since accurate slope estimates could not be obtained and represented 23.8% and 22.4% of all spikes for vehicle and ADP, respectively. [file Image_2.JPEG]
